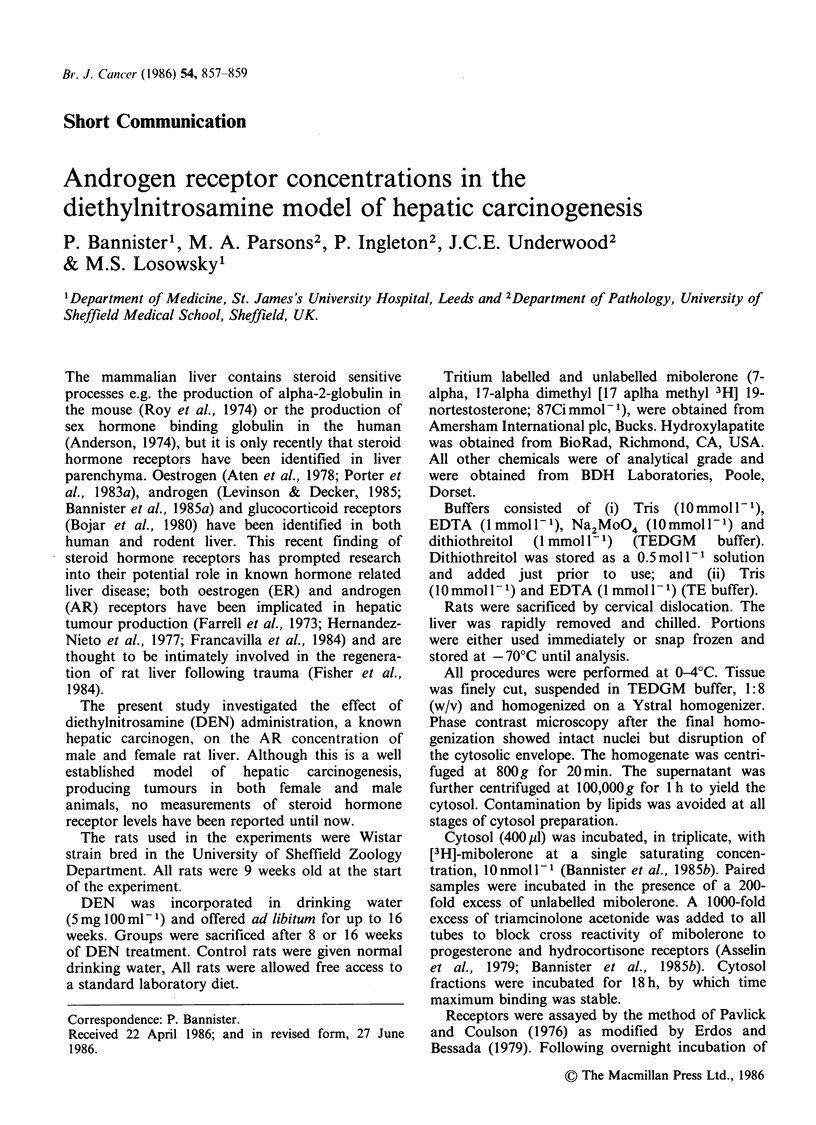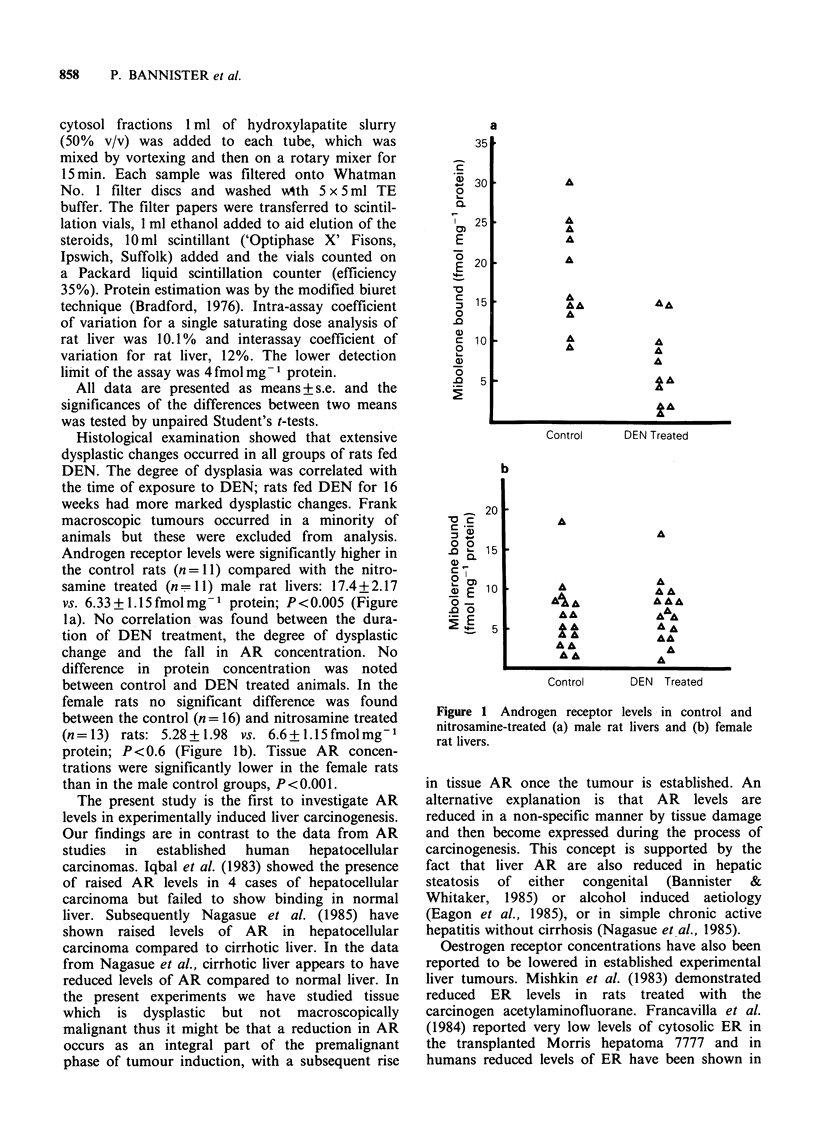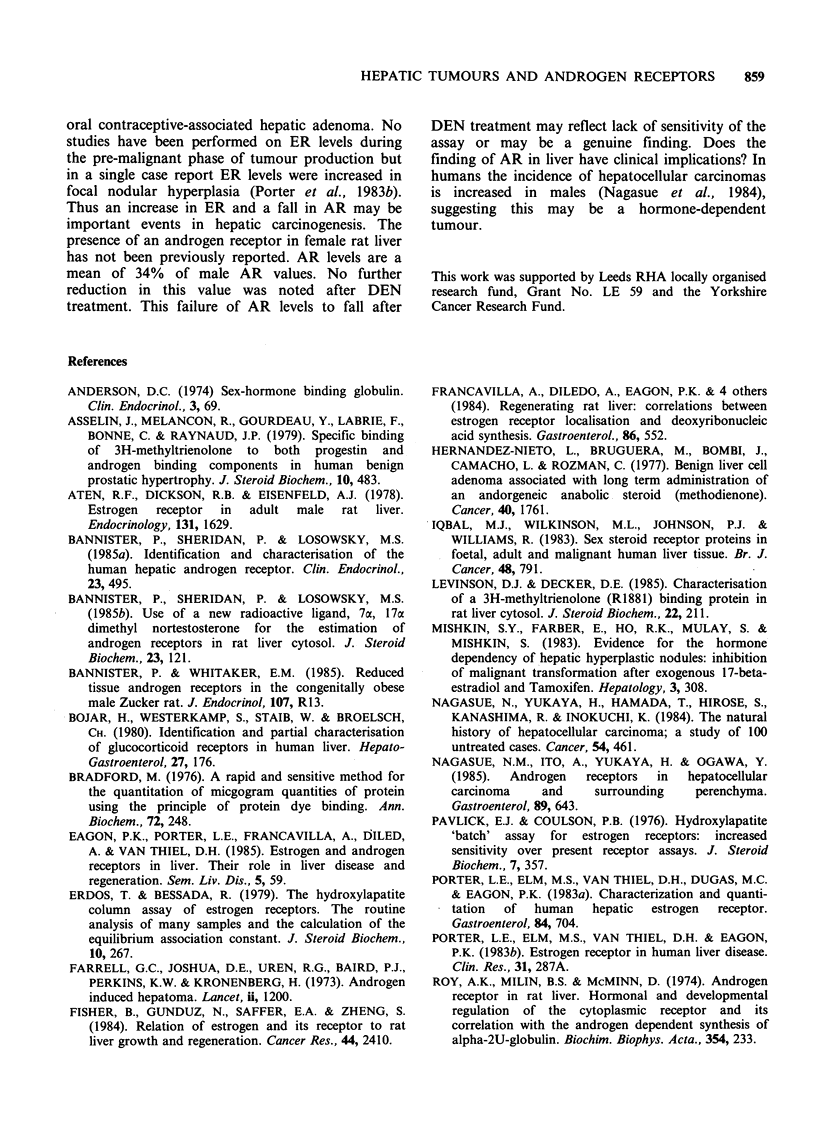# Androgen receptor concentrations in the diethylnitrosamine model of hepatic carcinogenesis.

**DOI:** 10.1038/bjc.1986.252

**Published:** 1986-11

**Authors:** P. Bannister, M. A. Parsons, P. Ingleton, J. C. Underwood, M. S. Losowsky


*Br. J. Cancer* (1986) **54**, 857–859

### Short Communication

### Androgen receptor concentrations in the diethylnitrosamine model of hepatic carcinogenesis

P. Bannister[1], M. A. Parsons[2], P. Ingleton[2], J.C.E. Underwood[2] & M.S. Losowsky[1]

[1]*Department of Medicine, St. James's University Hospital, Leeds and* [2]*Department of Pathology, University of Sheffield Medical School, Sheffield, UK.*

The mammalian liver contains steroid sensitive processes e.g. the production of alpha-2-globulin in the mouse (Roy *et al.*, 1974) or the production of sex hormone binding globulin in the human (Anderson, 1974), but it is only recently that steroid hormone receptors have been identified in liver parenchyma. Oestrogen (Aten *et al.*, 1978; Porter *et al.*, 1983*a*), androgen (Levinson & Decker, 1985; Bannister *et al.*, 1985*a*) and glucocorticoid receptors (Bojar *et al.*, 1980) have been identified in both human and rodent liver. This recent finding of steroid hormone receptors has prompted research into their potential role in known hormone related liver disease; both oestrogen (ER) and androgen (AR) receptors have been implicated in hepatic tumour production (Farrell *et al.*, 1973; Hernandez-Nieto *et al.*, 1977; Francavilla *et al.*, 1984) and are thought to be intimately involved in the regeneration of rat liver following trauma (Fisher *et al.*, 1984).

The present study investigated the effect of diethylnitrosamine (DEN) administration, a known hepatic carcinogen, on the AR concentration of male and female rat liver. Although this is a well established model of hepatic carcinogenesis, producing tumours in both female and male animals, no measurements of steroid hormone receptor levels have been reported until now.

The rats used in the experiments were Wistar strain bred in the University of Sheffield Zoology Department. All rats were 9 weeks old at the start of the experiment.

DEN was incorporated in drinking water ($5\,mg\,100\,ml^{-1}$) and offered *ad libitum* for up to 16 weeks. Groups were sacrificed after 8 or 16 weeks of DEN treatment. Control rats were given normal drinking water, All rats were allowed free access to a standard laboratory diet.

Correspondence: P. Bannister.
Received 22 April 1986; and in revised form, 27 June 1986.

Tritium labelled and unlabelled mibolerone (7-alpha, 17-alpha dimethyl [17 aplha methyl $^3H$] 19-nortestosterone; $87\,Ci\,mmol^{-1}$), were obtained from Amersham International plc, Bucks. Hydroxylapatite was obtained from BioRad, Richmond, CA, USA. All other chemicals were of analytical grade and were obtained from BDH Laboratories, Poole, Dorset.

Buffers consisted of (i) Tris ($10\,mmol\,l^{-1}$), EDTA ($1\,mmol\,l^{-1}$), $Na_2MoO_4$ ($10\,mmol\,l^{-1}$) and dithiothreitol ($1\,mmol\,l^{-1}$) (TEDGM buffer). Dithiothreitol was stored as a $0.5\,mol\,l^{-1}$ solution and added just prior to use; and (ii) Tris ($10\,mmol\,l^{-1}$) and EDTA ($1\,mmol\,l^{-1}$) (TE buffer).

Rats were sacrificed by cervical dislocation. The liver was rapidly removed and chilled. Portions were either used immediately or snap frozen and stored at $-70°C$ until analysis.

All procedures were performed at 0–4°C. Tissue was finely cut, suspended in TEDGM buffer, 1:8 (w/v) and homogenized on a Ystral homogenizer. Phase contrast microscopy after the final homogenization showed intact nuclei but disruption of the cytosolic envelope. The homogenate was centrifuged at 800 *g* for 20 min. The supernatant was further centrifuged at 100,000 *g* for 1 h to yield the cytosol. Contamination by lipids was avoided at all stages of cytosol preparation.

Cytosol ($400\,\mu l$) was incubated, in triplicate, with [$^3H$]-mibolerone at a single saturating concentration, $10\,nmol\,l^{-1}$ (Bannister *et al.*, 1985*b*). Paired samples were incubated in the presence of a 200-fold excess of unlabelled mibolerone. A 1000-fold excess of triamcinolone acetonide was added to all tubes to block cross reactivity of mibolerone to progesterone and hydrocortisone receptors (Asselin *et al.*, 1979; Bannister *et al.*, 1985*b*). Cytosol fractions were incubated for 18 h, by which time maximum binding was stable.

Receptors were assayed by the method of Pavlick and Coulson (1976) as modified by Erdos and Bessada (1979). Following overnight incubation of

© The Macmillan Press Ltd., 1986

cytosol fractions 1 ml of hydroxylapatite slurry (50% v/v) was added to each tube, which was mixed by vortexing and then on a rotary mixer for 15 min. Each sample was filtered onto Whatman No. 1 filter discs and washed with 5 × 5 ml TE buffer. The filter papers were transferred to scintillation vials, 1 ml ethanol added to aid elution of the steroids, 10 ml scintillant ('Optiphase X' Fisons, Ipswich, Suffolk) added and the vials counted on a Packard liquid scintillation counter (efficiency 35%). Protein estimation was by the modified biuret technique (Bradford, 1976). Intra-assay coefficient of variation for a single saturating dose analysis of rat liver was 10.1% and interassay coefficient of variation for rat liver, 12%. The lower detection limit of the assay was 4 fmol $mg^{-1}$ protein.

All data are presented as means ± s.e. and the significances of the differences between two means was tested by unpaired Student's *t*-tests.

Histological examination showed that extensive dysplastic changes occurred in all groups of rats fed DEN. The degree of dysplasia was correlated with the time of exposure to DEN; rats fed DEN for 16 weeks had more marked dysplastic changes. Frank macroscopic tumours occurred in a minority of animals but these were excluded from analysis. Androgen receptor levels were significantly higher in the control rats ($n = 11$) compared with the nitrosamine treated ($n = 11$) male rat livers: $17.4 \pm 2.17$ *vs.* $6.33 \pm 1.15$ fmol $mg^{-1}$ protein; $P < 0.005$ (Figure 1a). No correlation was found between the duration of DEN treatment, the degree of dysplastic change and the fall in AR concentration. No difference in protein concentration was noted between control and DEN treated animals. In the female rats no significant difference was found between the control ($n = 16$) and nitrosamine treated ($n = 13$) rats: $5.28 \pm 1.98$ *vs.* $6.6 \pm 1.15$ fmol $mg^{-1}$ protein; $P < 0.6$ (Figure 1b). Tissue AR concentrations were significantly lower in the female rats than in the male control groups, $P < 0.001$.

The present study is the first to investigate AR levels in experimentally induced liver carcinogenesis. Our findings are in contrast to the data from AR studies in established human hepatocellular carcinomas. Iqbal *et al.* (1983) showed the presence of raised AR levels in 4 cases of hepatocellular carcinoma but failed to show binding in normal liver. Subsequently Nagasue *et al.* (1985) have shown raised levels of AR in hepatocellular carcinoma compared to cirrhotic liver. In the data from Nagasue *et al.*, cirrhotic liver appears to have reduced levels of AR compared to normal liver. In the present experiments we have studied tissue which is dysplastic but not macroscopically malignant thus it might be that a reduction in AR occurs as an integral part of the premalignant phase of tumour induction, with a subsequent rise

**Figure 1** Androgen receptor levels in control and nitrosamine-treated (a) male rat livers and (b) female rat livers.

in tissue AR once the tumour is established. An alternative explanation is that AR levels are reduced in a non-specific manner by tissue damage and then become expressed during the process of carcinogenesis. This concept is supported by the fact that liver AR are also reduced in hepatic steatosis of either congenital (Bannister & Whitaker, 1985) or alcohol induced aetiology (Eagon *et al.*, 1985), or in simple chronic active hepatitis without cirrhosis (Nagasue *et al.*, 1985).

Oestrogen receptor concentrations have also been reported to be lowered in established experimental liver tumours. Mishkin *et al.* (1983) demonstrated reduced ER levels in rats treated with the carcinogen acetylaminofluorane. Francavilla *et al.* (1984) reported very low levels of cytosolic ER in the transplanted Morris hepatoma 7777 and in humans reduced levels of ER have been shown in oral contraceptive-associated hepatic adenoma. No studies have been performed on ER levels during the pre-malignant phase of tumour production but in a single case report ER levels were increased in focal nodular hyperplasia (Porter *et al.*, 1983*b*). Thus an increase in ER and a fall in AR may be important events in hepatic carcinogenesis. The presence of an androgen receptor in female rat liver has not been previously reported. AR levels are a mean of 34% of male AR values. No further reduction in this value was noted after DEN treatment. This failure of AR levels to fall after DEN treatment may reflect lack of sensitivity of the assay or may be a genuine finding. Does the finding of AR in liver have clinical implications? In humans the incidence of hepatocellular carcinomas is increased in males (Nagasue *et al.*, 1984), suggesting this may be a hormone-dependent tumour.

This work was supported by Leeds RHA locally organised research fund, Grant No. LE 59 and the Yorkshire Cancer Research Fund.

### References

ANDERSON, D.C. (1974) Sex-hormone binding globulin. *Clin. Endocrinol.*, **3**, 69.

ASSELIN, J., MELANCON, R., GOURDEAU, Y., LABRIE, F., BONNE, C. & RAYNAUD, J.P. (1979). Specific binding of 3H-methyltrienolone to both progestin and androgen binding components in human benign prostatic hypertrophy. *J. Steroid Biochem.*, **10**, 483.

ATEN, R.F., DICKSON, R.B. & EISENFELD, A.J. (1978). Estrogen receptor in adult male rat liver. *Endocrinology*, **131**, 1629.

BANNISTER, P., SHERIDAN, P. & LOSOWSKY, M.S. (1985*a*). Identification and characterisation of the human hepatic androgen receptor. *Clin. Endocrinol.*, **23**, 495.

BANNISTER, P., SHERIDAN, P. & LOSOWSKY, M.S. (1985*b*). Use of a new radioactive ligand, 7α, 17α dimethyl nortestosterone for the estimation of androgen receptors in rat liver cytosol. *J. Steroid Biochem.*, **23**, 121.

BANNISTER, P. & WHITAKER, E.M. (1985). Reduced tissue androgen receptors in the congenitally obese male Zucker rat. *J. Endocrinol*, **107**, R13.

BOJAR, H., WESTERKAMP, S., STAIB, W. & BROELSCH, CH. (1980). Identification and partial characterisation of glucocorticoid receptors in human liver. *Hepato-Gastroenterol*, **27**, 176.

BRADFORD, M. (1976). A rapid and sensitive method for the quantitation of micgogram quantities of protein using the principle of protein dye binding. *Ann. Biochem.*, **72**, 248.

EAGON, P.K., PORTER, L.E., FRANCAVILLA, A., DILED, A. & VAN THIEL, D.H. (1985). Estrogen and androgen receptors in liver. Their role in liver disease and regeneration. *Sem. Liv. Dis.*, **5**, 59.

ERDOS, T. & BESSADA, R. (1979). The hydroxylapatite column assay of estrogen receptors. The routine analysis of many samples and the calculation of the equilibrium association constant. *J. Steroid Biochem.*, **10**, 267.

FARRELL, G.C., JOSHUA, D.E., UREN, R.G., BAIRD, P.J., PERKINS, K.W. & KRONENBERG, H. (1973). Androgen induced hepatoma. *Lancet*, **ii**, 1200.

FISHER, B., GUNDUZ, N., SAFFER, E.A. & ZHENG, S. (1984). Relation of estrogen and its receptor to rat liver growth and regeneration. *Cancer Res.*, **44**, 2410.

FRANCAVILLA, A., DILEDO, A., EAGON, P.K. & 4 others (1984). Regenerating rat liver: correlations between estrogen receptor localisation and deoxyribonucleic acid synthesis. *Gastroenterol.*, **86**, 552.

HERNANDEZ-NIETO, L., BRUGUERA, M., BOMBI, J., CAMACHO, L. & ROZMAN, C. (1977). Benign liver cell adenoma associated with long term administration of an andorgeneic anabolic steroid (methodienone). *Cancer*, **40**, 1761.

IQBAL, M.J., WILKINSON, M.L., JOHNSON, P.J. & WILLIAMS, R. (1983). Sex steroid receptor proteins in foetal, adult and malignant human liver tissue. *Br. J. Cancer*, **48**, 791.

LEVINSON, D.J. & DECKER, D.E. (1985). Characterisation of a 3H-methyltrienolone (R1881) binding protein in rat liver cytosol. *J. Steroid Biochem.*, **22**, 211.

MISHKIN, S.Y., FARBER, E., HO, R.K., MULAY, S. & MISHKIN, S. (1983). Evidence for the hormone dependency of hepatic hyperplastic nodules: inhibition of malignant transformation after exogenous 17-beta-estradiol and Tamoxifen. *Hepatology*, **3**, 308.

NAGASUE, N., YUKAYA, H., HAMADA, T., HIROSE, S., KANASHIMA, R. & INOKUCHI, K. (1984). The natural history of hepatocellular carcinoma; a study of 100 untreated cases. *Cancer*, **54**, 461.

NAGASUE, N.M., ITO, A., YUKAYA, H. & OGAWA, Y. (1985). Androgen receptors in hepatocellular carcinoma and surrounding perenchyma. *Gastroenterol*, **89**, 643.

PAVLICK, E.J. & COULSON, P.B. (1976). Hydroxylapatite 'batch' assay for estrogen receptors: increased sensitivity over present receptor assays. *J. Steroid Biochem.*, **7**, 357.

PORTER, L.E., ELM, M.S., VAN THIEL, D.H., DUGAS, M.C. & EAGON, P.K. (1983*a*). Characterization and quantitation of human hepatic estrogen receptor. *Gastroenterol*, **84**, 704.

PORTER, L.E., ELM, M.S., VAN THIEL, D.H. & EAGON, P.K. (1983*b*). Estrogen receptor in human liver disease. *Clin. Res.*, **31**, 287A.

ROY, A.K., MILIN, B.S. & MCMINN, D. (1974). Androgen receptor in rat liver. Hormonal and developmental regulation of the cytoplasmic receptor and its correlation with the androgen dependent synthesis of alpha-2U-globulin. *Biochim. Biophys. Acta.*, **354**, 233.